# Supplementary material for: Default mode network activation at task switches reflects mental task-set structure
Source: Imaging Neurosci (Camb). 2025 Mar 24;3:imag_a_00515. doi: 10.1162/imag_a_00515 (PMC12319820; doi:10.1162/imag_a_00515)
Supplement: Supplementary Material [file imag_a_00515-supp.pdf]

Supplementary Materials

Supplementary Methods

For functional connectivity analyses, a separate GLM was created for each participant, similar to the GLM in the primary analysis, but with a separate regressor for each trial. A first analysis examined within-network connectivity, between the midline frontal and parietal regions of the Core DMN. A second analysis examined connectivity between the whole Core DMN and the multiple demand network (MDN). The frontoparietal MDN ROI was taken from Mitchell *et al.* (2016) based on data from Fedorenko *et al.* (2013). In each case, we used beta-series regression (Rissman et al., 2004) to quantify functional connectivity for each condition of interest, using trial-wise activity estimated from the GLM. For each subject, and each condition, we calculated the partial correlation between the activation vectors of each pair of ROIs, while controlling for the global activity averaged across the whole brain. Then, Fisher transformation was applied to the correlation values, resulting in one correlation value (Fisher's z) per subject, per pair of ROIs, for each type of task switch condition. The following comparison of conditions followed a similar approach to the primary analyses of activation amplitude in the main text. To assess the effects of task condition, first a one-way ANOVA was conducted across the six task switch conditions. Then, to test whether the within-domain and between-domain switch conditions differed as a function of current task complexity or instructional complexity, a three-way ANOVA included factors of condition (within-group-within-domain and within-group-between-domain), crossed with the two modulating factors of current task-set complexity (two domains, four domains) and instructed order (learnt first, learnt second).

Supplementary Results

Within-network functional connectivity between the two midline regions comprising the Core DMN subnetwork, is plotted in Supplementary Figure 1. A one-way ANOVA with the six task conditions (task-repeat, within-group within domain, within-group between-domain, between-group between-domain, restart and rest) showed expected significantly positive connectivity overall, as shown by the intercept ($F_{(1,35)}$ =58.67, $p<0.01$, BF_{10} =1.50 x 10^6), as well as a significant effect of condition ($F_{(5,175)}$ =4.86, $p<0.01$, BF_{10} =3.31), driven by slightly stronger connectivity during the rest and restart conditions. Building on the primary analyses in the main text, we were most interested in whether there was a difference between the within-domain switches and the between-domain switches, and whether this depended on current task complexity or instructional complexity. However, a three-way ANOVA with factors of condition (within-group-within-domain and within-group-between-domain), crossed with the two modulating factors of current task-set complexity (two domains, four domains) and instructed order (learnt first, learnt second), showed no significant effect of condition ($F_{(1,35)}$ =0.84, $p=0.36$, BF_{10}=0.32), or any interaction of condition with domain number or instructed order ($F_{(1,35)}$ <0.68, $p>0.41$, BF_{10}<0.29). There was a significant effect of domain

number ($F_{(1,35)}$ =6.54, p<0.02, BF_{10} =3.36), with lower connectivity in the four domain runs, but no main effect of instructed order ($F_{(1,35)}$ =0.25, p=0.62, BF_{10}=0.24).

Next, a similar set of analyses examined functional connectivity between the Core DMN subnetwork and the MD network (Supplementary Figure 2). A one-way ANOVA showed an overall anti-correlation, as expected (Fox et al., 2005), shown by the intercept ($F_{(1,35)}$ =210.37, p<0.01, BF_{10} =7.37 x 10^{12}), with no significant effect of condition ($F_{(5,175)}$ =2.26, p=0.07, BF_{10}=0.01). The three-way ANOVA with factors of condition (within-group-within-domain and within-group-between-domain), crossed with the two modulating factors of current task-set complexity (two domains, four domains) and instructed order (learnt first, learnt second) showed no significant main effect or interaction for any of the factors ($F_{(1,35)}$ < 1.09, p>0.15, BF_{10}< 0.54).

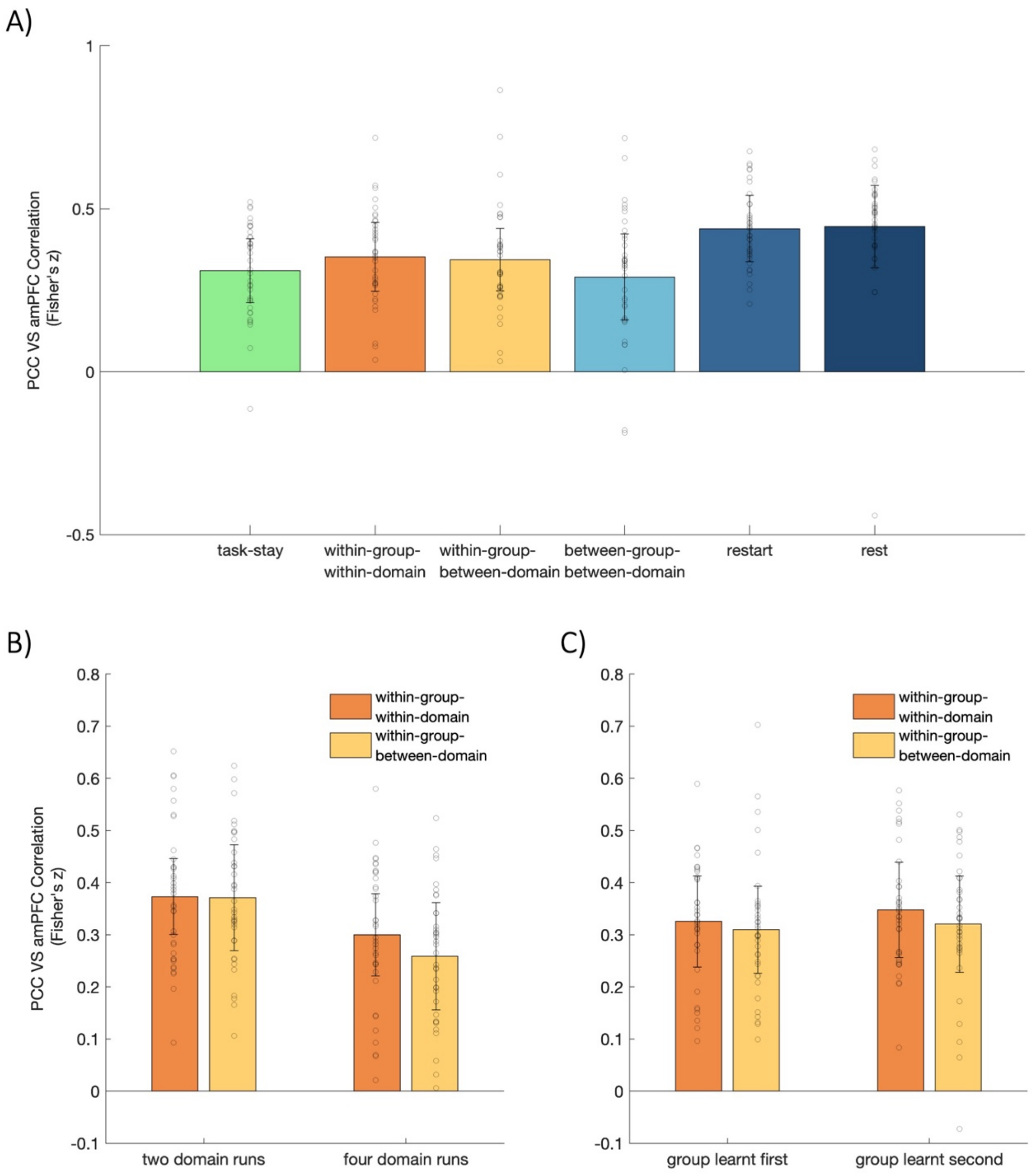

Supplementary Figure 1. A) Functional connectivity between midline frontal and parietal sub-regions of the Core DMN subnetwork, for each task switch condition. In the lower panels, connectivity for the within-group within- and between-domain switch conditions is split by domain number (B) and by instructed order (C). Error bars indicate between-subject 95% confidence intervals, and dots show data for individual participants, after removal of between-participant variance (Loftus & Masson, 1994).

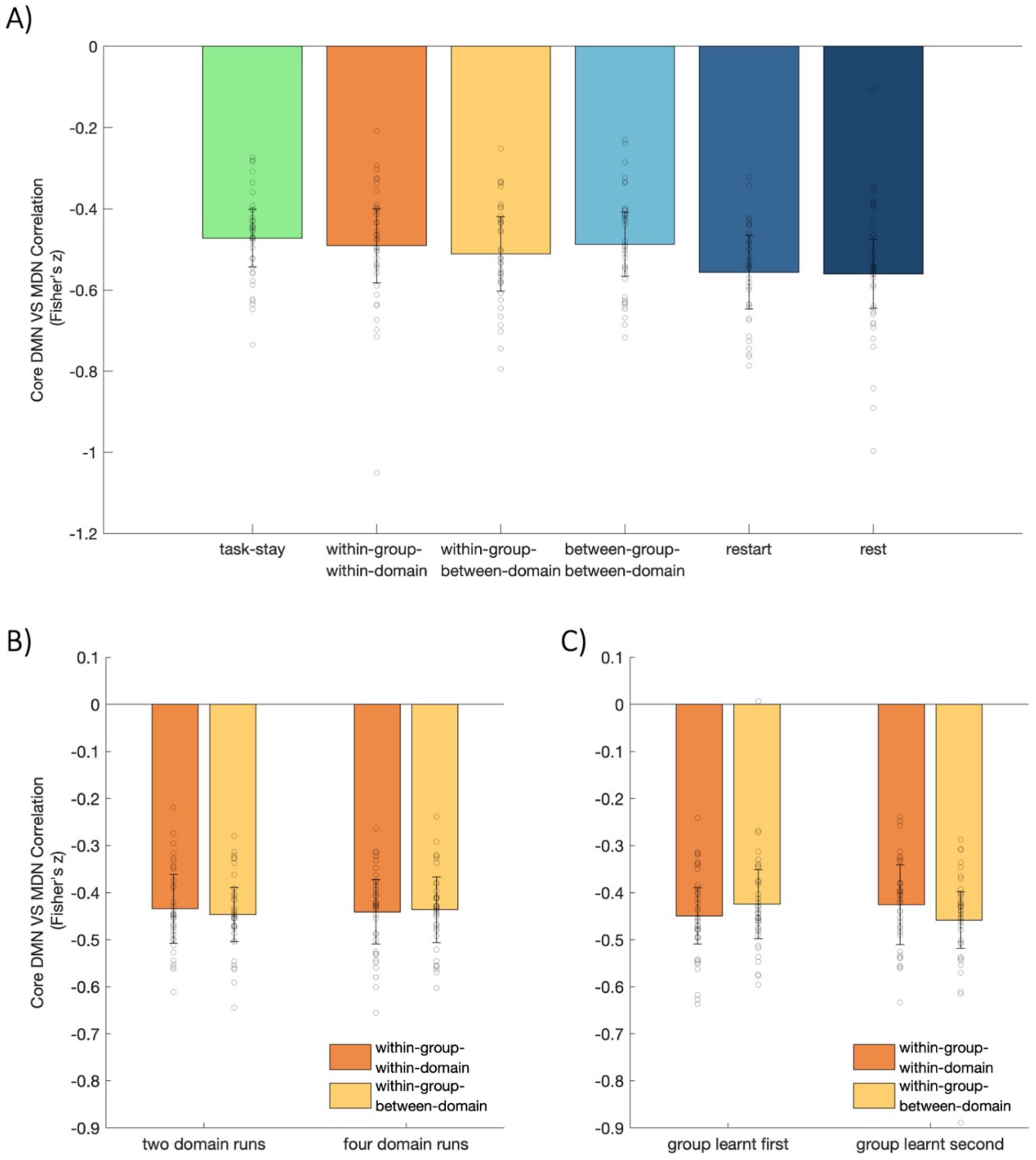

Supplementary Figure 2. A) Functional connectivity between the Core DMN subnetwork and the MD network, for each task switch condition. In the lower panels, connectivity for the within-group within- and between-domain switch conditions is split by domain number (B) and by instructed order (C). Error bars indicate between-subject 95% confidence intervals, and dots show data for individual participants, after removal of between-participant variance (Loftus & Masson, 1994).

Supplementary References

Fedorenko E, Duncan J, Kanwisher N (2013) Broad domain generality in focal regions of frontal and parietal cortex. Proc Natl Acad Sci U S A 110:16616-16621.

Fox MD, Snyder AZ, Vincent JL, Corbetta M, Van Essen DC, Raichle ME (2005) The human brain is intrinsically organized into dynamic, anticorrelated functional networks. Proceedings of the National Academy of Sciences of the United States of America 102:9673-9678.

Mitchell DJ, Bell AH, Buckley MJ, Mitchell AS, Sallet J, Duncan J (2016) A Putative Multiple-Demand System in the Macaque Brain. J Neurosci 36:8574-8585.

Rissman J, Gazzaley A, D'Esposito M (2004) Measuring functional connectivity during distinct stages of a cognitive task. Neuroimage 23:752-763.
